# Nested Machine Learning Facilitates Increased Sequence Content for Large-Scale Automated High Resolution Melt Genotyping

**DOI:** 10.1038/srep19218

**Published:** 2016-01-18

**Authors:** Stephanie I. Fraley, Pornpat Athamanolap, Billie J. Masek, Justin Hardick, Karen C. Carroll, Yu-Hsiang Hsieh, Richard E. Rothman, Charlotte A. Gaydos, Tza-Huei Wang, Samuel Yang

**Affiliations:** 1 Bioengineering, The University of California San Diego, La Jolla, California, 92093, USA; 2 Biomedical Engineering, The Johns Hopkins University, Baltimore, Maryland, 21218, USA; 3 Emergency Medicine, The Johns Hopkins University, Baltimore, Maryland, 21218, USA; 4 Infectious Disease, Medicine, The Johns Hopkins University, Baltimore, Maryland, 21218, USA; 5 Medical Microbiology, Pathology, The Johns Hopkins University, Baltimore, Maryland, 21218, USA; 6 Mechanical Engineering, The Johns Hopkins University, Baltimore, Maryland, 21218, USA; 7 Emergency Medicine, Stanford University, Stanford, California, 94305, USA

## Abstract

High Resolution Melt (HRM) is a versatile and rapid post-PCR DNA analysis technique primarily used to differentiate sequence variants among only a few short amplicons. We recently developed a one-vs-one support vector machine algorithm (OVO SVM) that enables the use of HRM for identifying numerous short amplicon sequences automatically and reliably. Herein, we set out to maximize the discriminating power of HRM + SVM for a single genetic locus by testing longer amplicons harboring significantly more sequence information. Using universal primers that amplify the hypervariable bacterial 16 S rRNA gene as a model system, we found that long amplicons yield more complex HRM curve shapes. We developed a novel nested OVO SVM approach to take advantage of this feature and achieved 100% accuracy in the identification of 37 clinically relevant bacteria in Leave-One-Out-Cross-Validation. A subset of organisms were independently tested. Those from pure culture were identified with high accuracy, while those tested directly from clinical blood bottles displayed more technical variability and reduced accuracy. Our findings demonstrate that long sequences can be accurately and automatically profiled by HRM with a novel nested SVM approach and suggest that clinical sample testing is feasible with further optimization.

High Resolution Melt (HRM) is a simple but powerful technique that holds great promise as a rapid and accessible DNA sequence identification method because it takes place as a single-tube process, is performed within about ten minutes directly after PCR, and has become a standard functionality available on most real-time PCR platforms. Many small-scale genotyping assays aimed at differentiating a limited set of target sequences have been developed with this technology. These have almost exclusively used amplicons less than ~300 bp in length, likely due to the fact that amplification of short amplicons is more reliable and typically generates a simple sigmoidal loss of fluorescence curve upon heating (i.e. a melt curve) with a single derivative peak defined as the melting temperature (Tm), which is highly sensitive to sequence differences^1^,^2^,^3^. Many factors contribute to the uniqueness of a sequence’s Tm, including G-C content, amplicon length, and location of variation. Nonetheless, short-length amplicons tend to produce a Tm within a relatively narrow temperature range (~4 °C in our previous work^4^,^5^,^6^,^7^,^8^,^9^,^10^) of the full melt spectrum (i.e. 50–95 °C). This represents a major technical challenge if HRM is to be expanded for large-scale genotyping of potentially thousands of sequences. Even if the ideal accuracy of 0.01 °C were achieved reproducibly, a 4 °C Tm range could only distinguish up to 400 targets. Reliance on Tm alone to differentiate varied sequence targets would require extremely reproducible Tms, with obvious limitations. Our goal here was to develop strategies for using HRM to accomplish larger-scale, reliable DNA sequence identification. Since nucleic acid melting interactions become more complex for longer stretches of sequence^1^,^2^,^11^,^12^, we hypothesized that long amplicons could produce additional distinguishing melt features beyond Tm alone. In addition, many genotyping applications require sequence stretches of a thousand base pairs or more to identify key sequence variants or relevant combinations of variations. For example, bacterial identification applications require sequence information spanning thousands of base pairs and multiple hypervariable regions of the bacterial 16 S rRNA genetic loci (16 S).

Sequence analysis of the 16 S gene has become the gold standard for phylogenetic and taxonomic identification of many human pathogens. This is because (1) the 16 S gene is present in almost all bacteria; (2) the overall function of the 16 S gene has remained conserved over time, yet non-functional sequence changes permit capture of phylogenetic divergence and evolution; and (3) the long length of the 16 S gene, (1500 bp) generally containing nine hypervariable regions, provides sufficient sequence informatics to enable genus and often species and subspecies level differentiation of organisms^13^. Although the steady rise in the accessibility of sequencing technologies has facilitated their increased use for more routine identification of bacteria via the 16 S gene, the multi-step hands-on processing requirements, long turn-around times, high costs, and inability to achieve read lengths covering long stretches remain limiting^13^,^14^,^15^,^16^,^17^. This is especially true for very dilute and/or heterogeneous samples involved in many clinical and research scenarios^18^,^19^,^20^. Approaches that are more rapid and accessible and that enable long sequences to be analyzed in whole without reassembly are needed for more accurate and sensitive identification of bacteria using the 16 S rRNA gene, especially for those applications requiring a digital or single cell level of sensitivity^9^,^21^. Herein, the use of a single primer set producing a long amplicon was designed to meet this need.

We and others have previously designed HRM assays for 16 S-based identification using multiple separate amplification reactions and primer sets to interrogate short sequence stretches within a genetic loci^4^,^5^,^6^,^7^,^9^,^22^,^23^,^24^,^25^. This was successful for identifying a pure sample, but fails in non-pure samples where multiple genotypes present a mixture of distinct sequences contributing to a single melt curve^10^. To overcome this challenge, we subsequently developed Universal digital High Resolution Melt (U-dHRM) by diluting the starting material such that each genotype is distributed into its own digital PCR reaction and generates a pure melt curve^9^. However, this approach is still limited because the amplicon’s Tm is typically the only significant differentiating feature of a short amplicon melt curve. To increase the profiling breadth and sequence information generated from a single primer set and to test the ability of HRM to resolve variations in long amplicons, we here explore the use of a ~1000 bp amplicon covering six hypervariable regions in the 16 S genetic loci for HRM curve-based identification.

The breadth of HRM genotyping assays have also been limited because of run-to-run variability in HRM curve Tm, which requires standards for each expected amplicon sequence to be run alongside unknown samples in each test for reliable comparison. We previously showed that support vector machine learning (SVM) is capable of overcoming much of this problem, removing the need for in-run standards. Instead, melt curve standards are used only initially to train the algorithm for subsequent identification. Our previous one-vs-one (OVO) SVM algorithm enabled automated identification of sigmoidal short amplicon melt curves with excellent reliability (99.8% accuracy), and was more accurate that other classification methods including kNN, Naïve Bayes, PCA-LDA^4^. Here we explore a new nested OVO SVM approach to maximize the benefit of long amplicon curve shape complexity and advance HRM’s ability to accomplish large-scale profiling.

## Results

**Figure 1:**
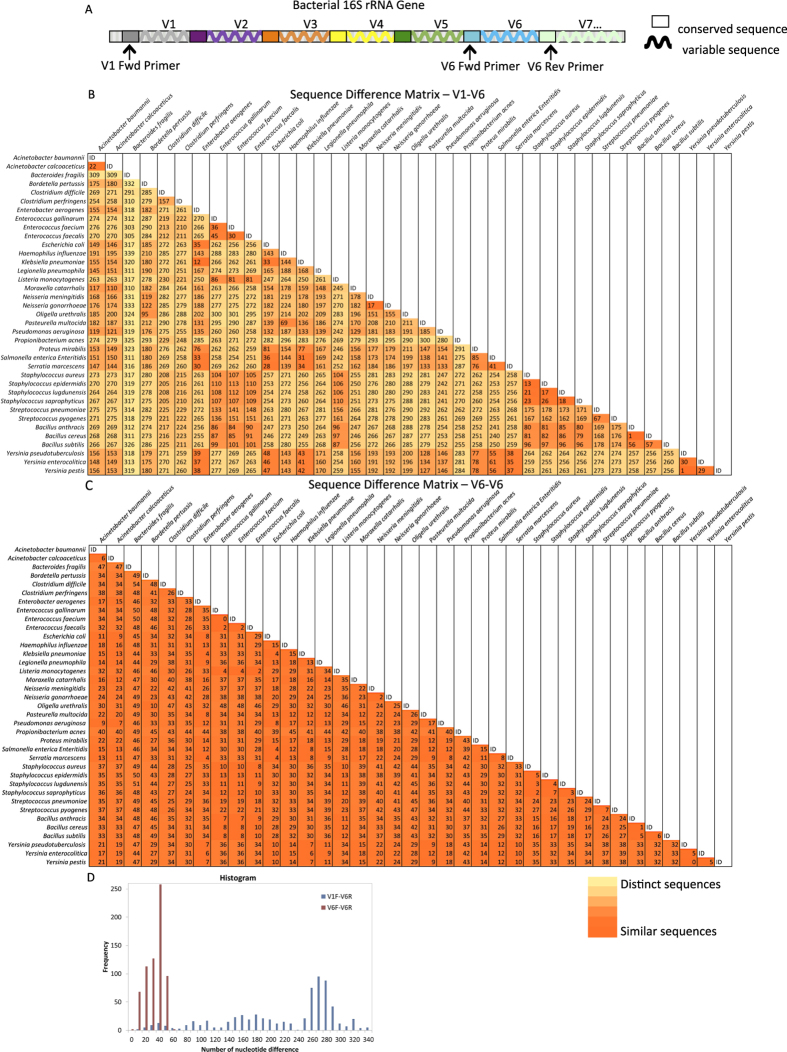
Bacterial 16 S ribosomal RNA (rRNA) gene map. (**A**) Diagram of bacterial 16 S rRNA gene showing seven (denoted V1-V7) of nine total hypervariable regions. Universal 16 S primers locate in conserved regions. Long amplicons include all six hypervariable regions (V1 through V6) by having universal forward primer (V1 Fwd) located before V1 and universal reverse primer (V6 Rev) located after V6. Short amplicons cover the V6 region by having universal forward (V6 Fwd) and reverse (V6 Rev) primers flanking the V6 region. (**B**) Sequence difference matrix of LAM V1-V6 amplicons of 37 organisms showing the number of nucleotide differences of the organisms compared with each other. Darker shading indicates fewer sequence differences, i.e. higher similarity, while light shading indicates greater sequence differences, i.e. lower similarity. (**C**) Sequence difference matrix of the V6 amplicon sequence among the same 37 organisms with same shading scale applied. (**D**) Histogram of sequence difference matrices in B & C showing a wider range of sequence differences among the one to one organism comparisons using the longer V1-V6 amplicon.

**Figure 2:**
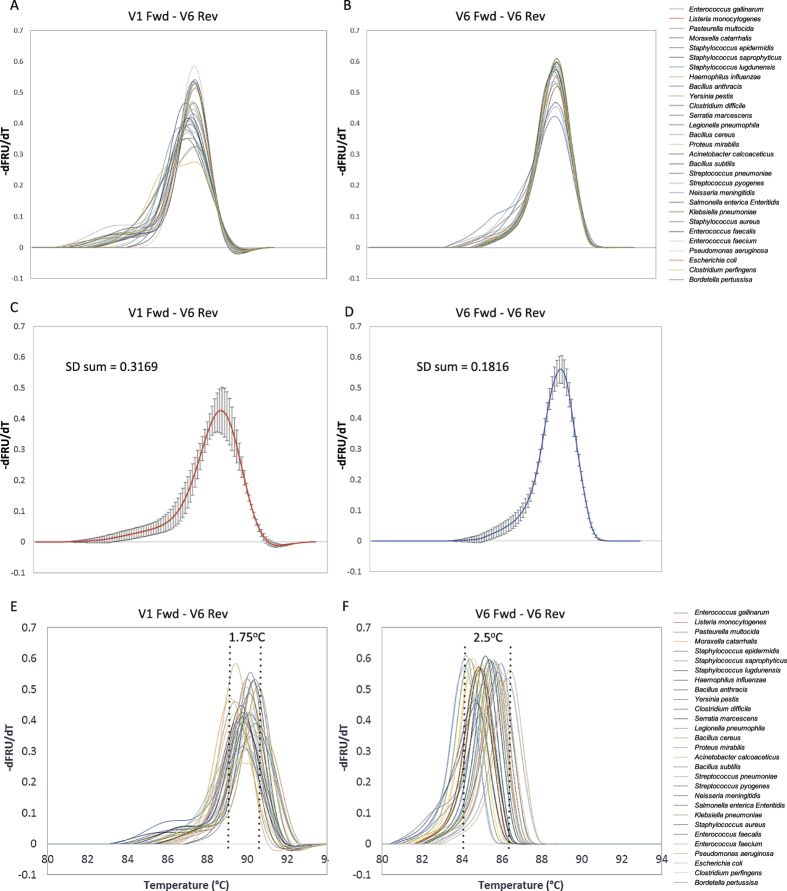
Melt curve shape diversity comparison between long and short amplicons of bacterial 16 S rRNA gene. (**A**,**B**) Derivative melt curves for the long and short amplicons respectively from 29 organisms aligned at their -dF/dT value equal to 0.1 on the right side for visual shape comparison. (**C**,**D**) The average of all LAM curves in A and B respectively with the sample standard deviation at each point. Summation of standard deviations shows 1.75 times greater shape diversity (S.D. = 0.3169) for the long amplicon than the short amplicon (S.D. = 0.1816). (**E**) LAM curves of 29 organisms with only temperature calibrator alignment, showing smaller peak Tm range (1.75 °C). (**F**) Short amplicon melt curves of 29 organisms with only temperature calibrator alignment, showing a wider peak Tm range (2.5 °C, 1.43 times the long amplicon range).

We hypothesized that long amplicon melting of the 16 S gene could identify as many as or more organisms as short amplicon melting of any single region alone, despite the amplicon being approximately six times longer than that typically used for HRM. First, we calculated the sequence differences in amplicons covering the hypervariable region V6 (Fig. 1A, ~130 bp) and compared this to amplicons covering the hypervariable regions V1-V6 (Fig. 1A, ~1000 bp) of 37 bacterial organisms. These organisms, spanning common pathogenic as well as commensal and near-neighbor bacteria across multiple phylogenetic families, genera, and species, were chosen based on their availability as sequence confirmed and phenotypically confirmed organisms. The V6 region was chosen as the short amplicon because it is the most variable and species specific single region of the 16 S gene^15^. Sequence difference heatmaps (Fig. 1B,C) and associated histogram (Fig. 1D) show that the range of sequence differences is much greater for the longer V1-V6 amplicon, as intended. Sequence differences among the 37 organisms ranged from 1–339 nucleotides for V1-V6 long amplicons and 0–54 nucleotides for V6-V6 short amplicons. To initially test whether these long amplicon sequence differences were resolvable by HRM, we generated experimental V6-V6 and V1-V6 melt curves for 29 of the organisms to compare their shape and Tm diversity. After aligning the curves at their derivative fluorescence value of 0.1 on their right side, it is visually clear that curve shape diversity is increased for long amplicon melt (LAM) curves compared to short amplicon curves (Fig. 2A,B). The average of the derivative melt curves for LAM and short amplicon curves and their standard deviations (Fig. 2C,D) show that the LAM curves are ~75% more diverse in shape, and this diversity is spread across more locations in the curves than for the short amplicons. However, the Tm values for short amplicon curves span a larger temperature range than LAM curves (Fig. 2E,F). For this comparison, temperature calibrator sequences were used, as in our previous work, to align curves for temperature axis accuracy^9^.

**Figure 3:**
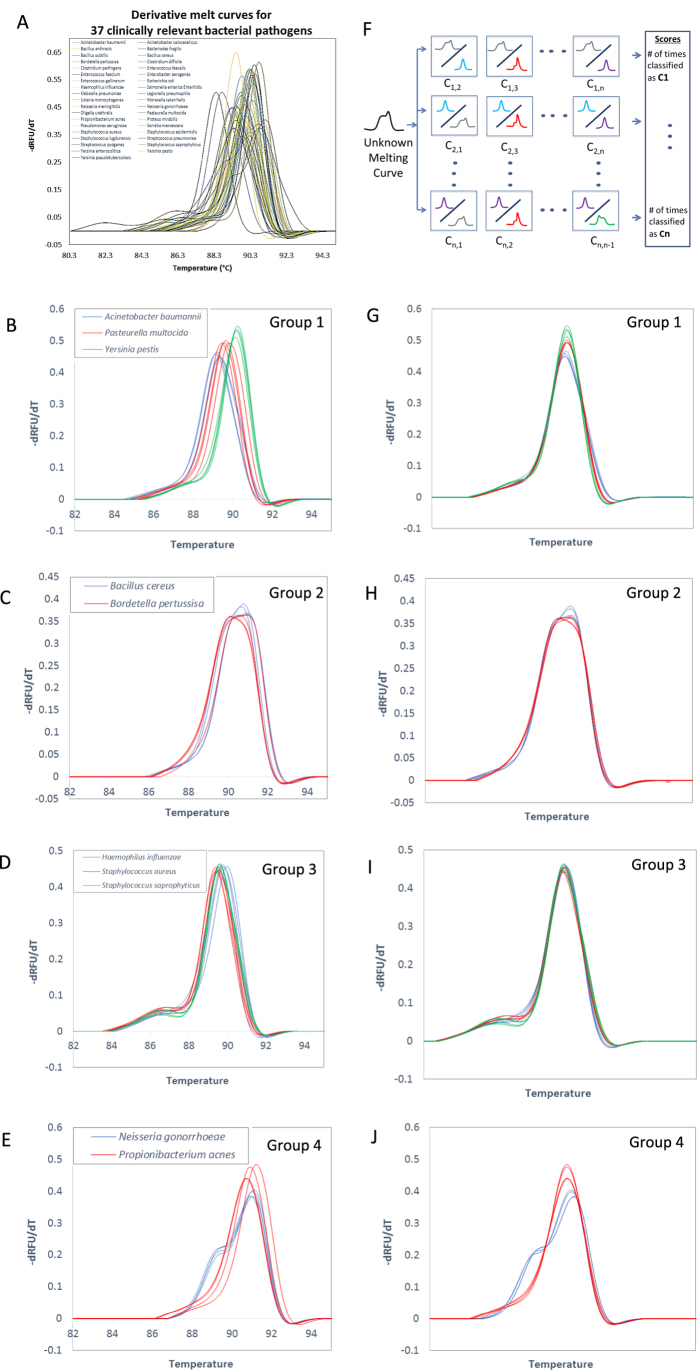
LAM curves with SVM melt curve classification. (**A**) LAM curves aligned by temperature calibrators of all 37 organisms showing LAM curve diversity. One representative curve from each organism. (**B–E**) Melt curves of organisms that are grouped together in SVM round one, which then proceed to SVM round two for further distinction. (**F**) One-vs.-one ensemble SVM scheme. An unknown curve is tested against each organism-pair SVM. An unknown curve will be called as organism C1 if organism C1 has the maximum score comparing to other organisms. (**G–J**) Derivative melt curves of each SVM round one group, (**B–E**), after shifting for SVM round two. This level classifies based on differences in shape without Tm consideration.

We next generated a full training library of LAM curves for the 37 organisms (see Methods). Figure 3A lists these organisms and shows a representative LAM curve for each. The full library included four replicate curves for each organism generated across two separate experiments. Given the superior performance of OVO SVM compared to other classification techniques for short amplicons^4^, we first tested our previously developed OVO SVM algorithm for its ability to uniquely and automatically identify LAM curves. Leave-One-Out Cross Validation (LOOCV) analysis with the original algorithm revealed that 10 of the 37 organism curves were not reliably identified despite curve shape differences that were distinguishable by visual examination. We then compared OVO SVM performance with one-vs-all (OVA) SVM and neural network (NN) approaches. OVA SVM was not able to reliably classify any of the organism training data (data not shown). However, several strategies for improving this approach were not tested, such as the incorporation of a threshold score value for classification. To test the utility of NN, we used feed-forward pattern recognition networks and varied the number of hidden layers and nodes in each layer to optimize performance. At best, NN achieved 89.2% accuracy. Several local optimums were identified, indicating that there was not enough training data for NN to fully learn the dataset (data not shown).

**Figure 4:**
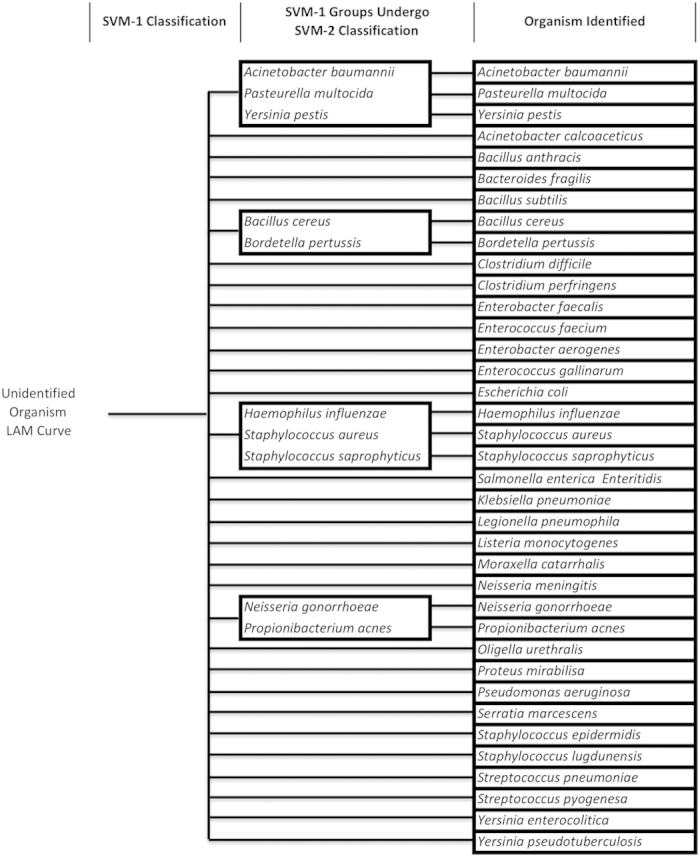
LAM nested SVM identification tree. Based on nested LOO SVM results of the training phase, the identification tree and testing phase SVM process is constructed. To test the identification of an experimental melt curve, round one SVM (left column) is used to classify the curve into either a group of organisms (middle column) or as an individual organism (right column). If the curve is identified to a group (middle column), it undergoes round two SVM within the group to be identified as an individual organism (right column). Middle column boxes correspond to groups that require further classification using SVM round two (Fig. 3). With this nested SVM process, all 37 organisms can be identified individually.

Given these findings, we set out to further advance our OVO SVM algorithm. Our finding that long amplicon sequence differences are manifested as changes in both curve shape and Tm prompted us to consider sequential rounds of OVO SVM, each targeting a particular type of curve change. To develop our new algorithm (Supplementary Fig. 1A), we used our original SVM algorithm^4^ trained on our library of 37 LAM curves to identify subgroups of organisms whose LAM curve Tms were highly similar, causing replicate curves to be occasionally misidentified (i.e. replicate organism curves were miscalled into multiple organism classifications). Organism curves that were miscalled were grouped with the organisms that they misidentified as (Fig. 3B–E). Sequential rounds of this re-grouping by LOOCV and SVM were completed until 100% accuracy was achieved among the groupings. Figure 3F shows the general process of SVM classification. Then, a second round of SVM was devised to differentiate within these groups based only on curve shape (see methods, Fig. 3G–J). The resulting nested SVM testing algorithm is depicted in Fig. 4 and is described in more detail in Supplementary Fig. 1B. Each box in Fig. 4 represents a classification group at different stages of the nested SVM testing algorithm process. Adding the second round of SVM analysis of LAM curve shape improved algorithm differentiation ability by 27%, enabling 100% resolution of all 37 organisms with LOOCV (Fig. 4).

We then performed blinded tests with new experimental LAM curves generated from different bacterial isolates of the organisms in our database to validate the ability of the finalized nested algorithm process and training data to identify bacteria correctly (see methods). These isolates were grown in a defined medium and their DNA was extracted from this medium and tested. Table 1 shows the results of organism inclusivity testing (different isolates of the same species), where 100% accuracy was achieved across 13 independent experimental test curves representing 4 different bacteria. To further test the algorithm’s performance, we collected 7 clinical samples from patients with confirmed and phenotypically identified bacteremia. Bacterial DNA was extracted directly from blood bottles and a 1:1000 dilution of the extracted DNA sample was used to generate 4–5 replicate LAM curves for testing. This dilution was chosen in an attempt to minimize the carryover of PCR inhibitory compounds from the blood bottle. Replicates were performed to assess the reproducibility of the melt curves within each sample. Table 2 shows the results of these clinical tests. Three out of 7 organisms were identified correctly in all of their replicate tests (P. aeruginosa, S. aureus, and B. fragilis). However, 3 of the remaining organisms were only identified correctly in 1–2 of their replicate tests and S. marcescens was not correctly identified in any of the replicate tests.

## Discussion

Using the hypervariable bacterial 16 S gene as a model system, we found that long amplicons (~1000 bp) generate HRM curves of greater shape complexity, occasionally with multiple Tm peaks, than short amplicons (<300 bp). For 37 clinically relevant bacteria, each with a unique 16 S gene sequence, we are able to automatically identify the bacteria by universally amplifying the gene, generating an HRM curve of the amplified sequence, and training our a nested OVO SVM algorithm to classify the melt curves. Previously, the separate amplification of three hypervariable regions in independent reactions (since their curves would overlap in a single reaction) was required for identification of these bacteria^10^. Our algorithm was designed as a hierarchical binary SVM, which classifies LAM curves by Tm and curve shape in sequential steps. This structure builds on our previous *in silico* success with OVO SVM for short amplicons while further taking advantage of the unique shape features of long amplicon melting curves. The choice of two layers was based on our observations that melt curve differences primarily manifest as shifts of the curve along the temperature axis (generally longer/more GC rich sequences are shifted to higher temperatures) and as changes in the shape of the curve (longer amplicons tend to produce complex, multi-inflection point melt curves compared to the sigmoidal shape of most short amplicons). Because experimental noise predominantly affects the reproducibility of the temperature axis values of any given melt curve, melt curves that are only slightly distinct in shape but largely overlapping in their temperature range are strongly affected by any temperature inaccuracies during the first round of OVO SVM classification. The addition of a temperature independent comparison of shape overcomes the temperature inaccuracies that tend to pull groups together. As we show, longer amplicons tend to have a more narrow range of melting temperatures than short amplicons, such that they are more likely to be affected by temperature inaccuracies.

Previously, we tested several approaches to classify short amplicon melting curves, included kNN, Naïve Bayes, PCA-LDA, and OVO SVM^4^. SVMs generally require fewer training examples than several other classification methods and are insensitive to the number of dimensions, even when the number of dimensions is greater than the number of samples. Also, SVMs guarantee that the best classification function is found by maximizing the margin between two classes. OVO SVM proved to be the most accurate for large-scale classification of short amplicon curves generated *in silico*. Yet, when we applied our original OVO SVM algorithm to ~1000 bp sequences for 16 S bacterial identification applications, our original algorithm did not perform as well as it did previously for the *in silico* short amplicon case. Adding a second round of OVO SVM after aligning curves independent of their temperature data, allowing them to be compared solely on curve shape, achieved 100% resolution of the training data set.

We also tested OVA SVM and NN approaches. With the same LAM training data, OVA SVM LOOCV resulted in none of the 4 × 37 organism curves being identified correctly. However, this method could likely be improved by the inclusion of more training data and the incorporation of a likelihood score (transform f-score into probability estimate) so that a threshold score value could be set for classification. NNs require more training examples and also require a large number of different parameters to be decided upon/optimized (e.g. number of layers, number of neurons per layer, number of training iterations, etc.) with no general and explicit method for choosing the parameters. NNs also have a tendency to overfit. Nonetheless, even with our relatively sparse training data set NN achieved 89.2% accuracy in LOOCV of the training set after some optimization (data not shown), but multiple local optimums were identified suggesting that more training data is needed for the NN to fully learn the data. Overall, our hierarchical OVO SMV proved to be the most robust classifier for LAM data.

Blinded testing of additional LAM curves generated from four different cultured isolates of organisms in our database demonstrated reliable identification across 2–4 replicate test curves each. Although this test did not cover all bacteria in our database, the results suggest that our assay is robust in these cases to strain-level variation and experimental variation. However, LAM curves generated from bacterial DNA that was extracted directly from clinical blood bottles demonstrated significant melt curve variation within individual samples. Three of the 7 clinical samples were correctly identified across all 5 replicates of the samples, but 4 clinical samples were inconsistently classified with 0–2 out of 5 replicates being correctly identified. We suspect that the known phenomena of extraction carryover of PCR inhibitors and/or chemicals that affect the melting process are primarily to blame. Further optimization of the assay for this sample type will be required, with training data being generated within the same sample background. It is important to note that the differentiation ability of any SVM classifier depends heavily on (1) the amount of training data available and (2) the number of classifications needed. Based on our previous findings^4^, it is likely that generating more replicate LAM training data will facilitate expansion in the size of our database while further increasing the accuracy of classification. In addition, segmenting the library into syndrome-based organisms (e.g. respiratory vs. urinary tract pathogens) could be a strategy for maintaining accuracy while increasing the scale of sequence differentiation ability.

A current limitation of our technique is categorization of bacteria in the face of phylogenetic novelty. Bacteria that are not represented in our database will still be classified as the organism in the database that has the most similar LAM curve. Creating a fully comprehensive database would be a challenge. However, several strategies for overcoming this limitation exist. For example, with sufficient volume of training data, SVM f-scores can be transformed into probability estimates to develop a confidence score for the classification accuracy of an unknown test curve^26^.

Previous reports have suggested that the ability of HRM to resolve single nucleotide differences among amplicons reduces dramatically with increases in amplicon length^4^. Our nested machine learning algorithm combined with LAM was able to accurately resolve all the sequence differences in this study, including a single nucleotide difference (C→A) positioned 70 nucleotides from the end of the V1-V6 amplicon (*B. anthracis* vs. *B. cereus*) and another single nucleotide difference (A→G) positioned 66 nucleotides from the end of the V1-V6 amplicon (*Y. pestis* vs. *Y. pseudotuberculosis*). Future expansion of our database will reveal any limitations to this level of sequence specificity. We anticipate that this will depend on the position of the variation within the amplicon and the class of the variation (A < – > T and G < – > C variations were not tested here). Nonetheless, given the results of this study, a strategy to overcome future specificity limitations could include amplifying an even longer stretch of the 16 S to cover all nine variable regions, adding even more sequence diversity to the amplicons.

The results of this study suggest that the melting of long amplicons coupled with machine learning techniques can provide a rigorous, rapid, and high-throughput identification technique for long continuous DNA sequence stretches. In addition to the application for microbial identification demonstrated here, our technique can potentially impact many other areas. It could be particularly useful for identifying DNA that is difficult to sequence due to repetitive nucleotides that hamper alignment of short read lengths, a limitation of many sequencing technologies. Because our technique is able to identify long sequence stretches, reassembly or combinations of multiple amplicons is avoided. This enables our LAM technique to scale to the digital/single cell format we previously developed, making it highly sensitive to low level targets and enabling it to be used for identification of heterogeneous genotypes (e.g. polymicrobial infection). Another potential impact of our work is its ability to inform models of DNA melting behavior. Finally, the clinical utility of this molecular microbiological approach as a promising diagnostic tool is currently under evaluation by our group.

## Methods

### Growth and DNA Extraction of Bacterial Isolates and Cell Lines

Thirty-seven clinically isolated or American Type Culture Collection (ATCC) bacterial strains (see Table 1) were grown on appropriate media, either Trypticase Soy Agar with 5% Sheep Blood (TSA II Agar, Becton Dickinson, Sparks, MD) or Chocolate II Agar with Hemoglobin and IsoVitaleX (GC II Agar, Becton Dickinson), at 37 °C for 24 hours prior to harvest and extraction. Anaerobic organisms were grown at 37 °C on appropriate growth media inside Bio-Bag Type C (Becton Dickinson) prior to harvest and extraction. Approximately 5–10 bacterial colonies were harvested from growth media and suspended in 500 μl of autoclaved, filtered (Corning Inc., Corning, NY), DNase (DNase I, Ambion, Life Technologies, Grand Island, NY) treated nuclease-free water (Ambion, Life Technologies). Suspensions were prepared in duplicate for each bacterial organism and centrifuged at 20,817 × g for 10 minutes. The supernatant was removed and bacterial pellets were pooled in a total volume of 200 μl of autoclaved, filtered, DNase treated nuclease-free water. DNA extraction was performed as previously described^9^.

Following DNA extraction, spectrophotometer readings were taken to assess the quality and quantity of the extracted DNA using the NanoDrop 1000 (Thermo Fisher Scientific, Waltham, MA). Extracted material was then diluted to a concentration of 1,000 copies of the 16 S rRNA gene per PCR reaction for each organism using the mass determined by spectrophotometer measurements and the known number of copies of the 16 S rRNA gene per organism genome (http://rrndb.umms.med.umich.edu/last accessed 12/20/2013 or http://www.ncbi.nlm.nih.gov/last accessed 12/20/2013).

### DNA Extraction from Clinical Blood Bottles

DNA was extracted from culture positive, phenotypically identified clinical blood bottles following methods previously developed^9^. Briefly, each blood bottle was mixed by inverting several times and then 500 μl was placed in a microcentrifuge tube and centrifuged for 20 s to precipitate solids. Two hundred microliters of the supernatant was used for DNA extraction as previously described^9^. The blood bottle DNA extract was serially diluted in 10-fold increments to 1:1000, and this dilution was used as input for universal 16 S PCR and LAM.

### PCR and High Resolution Melt Conditions for Library Generation and Inclusivity Testing

LAM reactions were performed in a 25 μl final volume and contained the following final concentrations: 1X PCR buffer (Taq PCR Core Kit, Qiagen, Venlo, Netherlands); 3.5 mM MgCl_2_ (Taq PCR Core Kit, Qiagen); 150 nM forward (V1F, 5′ GYGGCGNACGGGTGAGTAA 3′) and reverse (V6R 5′ AGCTGACGACANCCATGCA 3′) primers (Integrated DNA technologies, Coralville, IA); 50 nM low temperature calibrator^9^; 1X Evagreen (Biotium, Hayward, CA); 200 μM dNTPs (Taq Core PCR kit, Qiagen); 0.05 U/μl LD Taq Polymerase (Life Technologies, Carlsbad, CA), 2 μl genomic DNA and addition of autoclaved, filtered, DNase treated nuclease-free water to the final reaction volume. Reactions were covered with a 15 μl overlay of PCR grade mineral oil (Sigma Aldrich, St. Louis, MO), and cycling conditions were performed as previously described^9^ with the following modification, the extension step at 72 °C was for 90 seconds. Reactions were analyzed utilizing the LightScanner (BioFire Diagnostics, Salt Lake City, UT) as previously described^9^,^27^. Raw melt data files were exported and sent for further analysis utilizing the support vector machine learning algorithm.

All organisms were subjected to standard Sanger sequencing as previously described^9^ and the identity of each organism utilized in LAM reactions was confirmed by analyzing the generated sequence utilizing BLAST and the NCBI database of 16 S rRNA sequences.

### Sequence Analysis

The results of Sanger sequencing (typically 300–500 bp long) were used to choose the best matching database sequence in the NCBI database of 16 S sequences using BLAST. These sequences were collected and aligned with our primer sequences using ClustalOmega^28^, then truncated to the amplicon region only and realigned. Bioedit software^29^ was used to calculate the sequence difference matrices presented herein.

### Support Vector Machine Learning Algorithm Training and Nested Algorithm Development

We have developed a nested LAM curve classification algorithm consisting of two OVO ensemble SVM steps in order to classify 37 organisms (Fig. 3) using Matlab software. At each round, our ensemble SVM consists of N × (N-1)/2 binary SVMs where N represents the number of organisms or groups of organisms we need to classify (Fig. 3F). With the Least Square Method, the SVM function created a hyperplane separating the two organisms (binary) under comparison. To identify a melt curve, the curve was tested against all possible binary SVMs at a particular round and the score for each organism (1, 2, 3,…, N) was counted based on how many times the curve was called as that specific organism against every other organism. The organism with the highest score was the identification reported for that melt curve.

To develop and train the algorithm, we first created (37 × 36)/2 binary SVM classifiers (Fig. 3F, Supplementary Fig. 1A). Each organism was represented by 4 data points in the SVM feature space, which were derived from its 4 experimental training LAM curves. This was done by mapping each normalized negative derivative melt curve of 252 data points onto a single data point in a 252 dimensional feature space using the linear kernel function. Then the Leave One Out Cross Validation (LOOCV) method was used to group organisms whose melt curves were too similar to be discriminated reliably. LOOCV consisted of leaving one melt curve out of the dataset at a time as a testing curve and using the remaining three melt curves for training the SVM. When the testing melt curve from one organism was miscalled as another organism, the two organisms were grouped together. After this, 37 organisms were grouped into 32 distinct organism groups. Then this process was then repeated, where each binary SVM was performed between two groups of organisms from the first grouping round. For example, if after the first grouping round, organisms A, B and C were grouped together as group 1 and organisms X and Y were grouped together as group 2, then the second grouping round between groups 1 and 2 would be trained with 15 total curves—9 curves from group 1 and 6 curves from group 2 (3 curves from each organism). As a consequence of the second round group-based-SVM and LOOCV, organisms were again grouped with miscalls yielding 31 final groups. To improve resolution within the final groupings, a second round of group-based SVM and LOOCV was applied, but with the distinction of first performing temperature independent melt curve alignment. That is, within each of the groups, each normalized derivative melt curve was aligned at the full width center at 60% of the height of the melting peak so that the melting curve shapes could be compared without considering temperature shifting. Then binary SVM trained with 3 aligned melt curves from each individual organism within a given group and LOOCV performed. Further classification within each group was achieved, yielding 100% classification accuracy in LOOCV.

### Nested Support Vector Machine Learning Algorithm and Inclusivity Testing

For an unknown melt curve, we applied the two sequential OVO SVM rounds using the organism groupings determined during the training and development phase (see previous section of Methods), as shown in Supplementary Fig. 1B and in Fig. 4. After data pre-processing of the unknown curve into a normalized and temperature calibrated derivative melt curve, it was classified with the first round of SVM among 32 groups. If the curve identified to groups 1–4 (Figs 3B–E and 4, middle column), it was further processed by aligning it with database melt curves of the organisms in that group at the center of full width at 60% max and then processed by round two SVM. Finally, the curve is identified as a single organism match.

## Additional Information

**How to cite this article**: Fraley, S. I. *et al*. Nested Machine Learning Facilitates Increased Sequence Content for Large-Scale Automated High Resolution Melt Genotyping. *Sci. Rep.* **6**, 19218; doi: 10.1038/srep19218 (2016).

## Supplementary Material

Supplementary Information

## Figures and Tables

**Table 1:** Nested SVM Algorithm Inclusivity Test Results – Isolates in Pure Culture.

No.	Organism	Number of replicates	Number of correct ID	% Accuracy
1	*Bacillus anthracis*	4	4	100
2	*Legionella pneumophila*	2	2	100
3	*Pseudomonas aeruginosa*	3	3	100
4	*Yersinia pestis*	4	4	100

*different bacterial isolates than those used for training curve generation.

**Table 2:** Nested SVM Algorithm Inclusivity Test Results – Clinical Blood Bottles.

No.	Organism	Number of replicates	Number of correct ID	% Accuracy
1	*Pseudomonas aeruginosa*	5	5	100
2	*Escherichia coli*	5	2	40
3	*Staphylococcus aureus*	5	5	100
4	*Serratia marcescens*	5	0	0
5	*Klebsiella pneumoniae*	5	1	20
6	*Bacteriodes fragilis*	5	5	100
7	*Enterococcus faecium*	4	1	25
