# Supplementary material for: Nested Machine Learning Facilitates Increased Sequence Content for Large-Scale Automated High Resolution Melt Genotyping
Source: Sci Rep. 2016 Jan 18;6:19218. doi: 10.1038/srep19218 (PMC4726007; doi:10.1038/srep19218)
Supplement: Supplementary Information [file srep19218-s1.doc]

**Nested Machine Learning Facilitates Increased Sequence Content for Large-Scale Automated High Resolution Melt Genotyping**

Stephanie I. Fraley^1^ŧ*†, Pornpat Athamanolap^2^†, Billie J. Masek^3,4^, Justin Hardick^3,4^, Karen C. Carroll^5^, Yu-Hsiang Hsieh^3^, Richard E. Rothman^3^, Charlotte A. Gaydos^4^, Tza-Huei Wang^2,6^, Samuel Yang^7^ŧ*

**Supplementary Figures:**

A

B


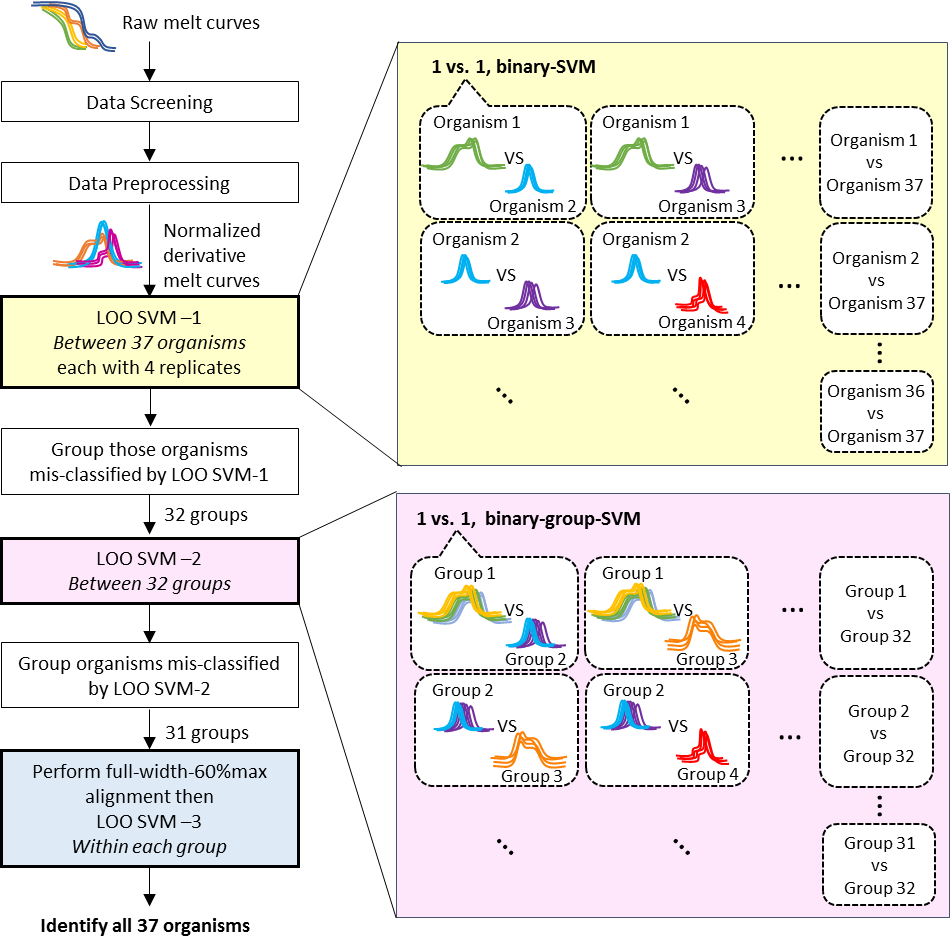

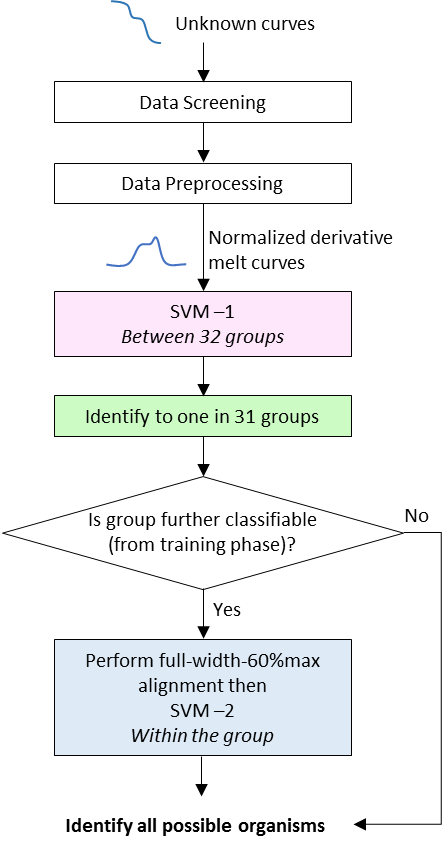


**Supplementary Figure 1. Process flowcharts for training and testing algorithms.** A) Process development and curve training phase for constructing machine learning classification module for 37 organisms with sequence-confirmed melt curves. Experimentally generated raw melt curves are quality screened, background subtracted, normalized, and classified with LOO round one SVM. Miscalled organisms are grouped together and LOO SVM round two is performed to test accuracy of classification between groups. Miscalled groups are grouped together again and LOO SVM round three is performed after curve alignment to test the ability to distinguish individual organisms within groups containing more than one organism. 100% classification accuracy is achieved. B) Testing phase. Each unknown curve goes through the same data pre-processing as in A and then is tested with SVM round one, classifying as one of the 31 organisms/groups of organisms resulting from the process in A. If it classifies as a group of organisms that could be further resolved by shape analysis, the curve is then aligned with the group and tested with SVM round two within the group. Based on this process, test curves can be accurately and automatically identified.
